# Risk of diabetes mellitus in physicians: a nationwide study in Taiwan

**DOI:** 10.1186/s12889-019-7403-z

**Published:** 2019-08-05

**Authors:** Shang-Gyu Lee, I-Jung Feng, Chien-Chin Hsu, Yi-Fong Wang, Chewn-Yi Yang, Jhi-Joung Wang, Jui-Yuan Chung, Chien-Cheng Huang

**Affiliations:** 1 Department of Endocrinology, Chi-Mei Medical Center, Tainan, Taiwan; 2 Department of Medical Research, Chi-Mei Medical Center, Tainan, Taiwan; 3 Department of Emergency Medicine, Chi-Mei Medical Center, 901 Zhonghua Road, Yongkang District, Tainan City, 710 Taiwan; 4 Department of Biotechnology, Southern Taiwan University of Science and Technology, Tainan, Taiwan; 5 Department of Leisure, Recreation and Tourism Management, Southern Taiwan University of Science and Technology, Tainan, Taiwan; 6 Allied AI Biomed Center, Southern Taiwan University of Science and Technology, Tainan, Taiwan; 7 Department of Emergency Medicine, Cathay General Hospital, 280 Renai Rd. Sec.4, Taipei, Taiwan; 8 Department of Senior Services, Southern Taiwan University of Science and Technology, Tainan, Taiwan; 9 Department of Environmental and Occupational Health, College of Medicine, National Cheng Kung University, Tainan, Taiwan

**Keywords:** Physician, Diabetes mellitus, Emergency physician, Surgeon

## Abstract

**Background:**

The heavy workload of physicians in Taiwan may contribute to poor lifestyles and increased risk for diabetes mellitus (DM). We conducted this study to determine the risk for DM among physicians in Taiwan.

**Methods:**

We used the Taiwan National Health Insurance Research Database to identify 28,440 physicians and 56,880 comparisons (general population) matched at a ratio of 1:2 by age and sex. Participants who had been diagnosed with DM before 2007 were excluded. We compared the risk for DM between physicians and comparisons by following up since 2007 to 2013. Comparisons among physician subgroups were also performed.

**Results:**

After adjustment for hypertension, hyperlipidemia, hyperuricemia, coronary artery disease, congestive heart failure, hyperthyroidism, hypothyroidism, and polycystic ovary syndrome, physicians had a lower risk for DM than the comparisons (adjusted odds ratio [AOR]: 0.75; 95% confidence interval [CI]: 0.68–0.82). In comparisons among physicians, emergency physicians (AOR: 2.21; 95% CI: 1.44–3.40) and surgeons (AOR: 1.26; 95% CI: 1.05–1.52) had a higher risk for DM than other specialists.

**Conclusions:**

This study found that physicians have a lower risk for DM than the general population and emergency physicians and surgeons have a higher risk for DM than other specialists. Thus, more attention should be paid to the occupational health of these doctors.

## Background

Diabetes mellitus (DM) is a global disease that shows a dramatic increase in prevalence every year. The global diabetic population is expected to reach 366 million in 2011 and 552 million in 2030 [1]. In Taiwan, an increase of over 70% in the total diabetic population was observed from 2000 to 2009 [2]. DM is associated with many subsequent complications, including retinopathy, nephropathy, neuropathy, cardiovascular diseases, and death, and greatly increases the burden of medical expenditures [3–7].

Physicians in Taiwan generally bear a heavy workload, as 50% of the country’s physicians work over 57 h per week, 34.5% work as many as 65 h per week, and approximately 10.6% require an average of 21 extra hours on top of the work average of other professions [8]. Many physicians, such as emergency physicians, surgeons, critical-care specialists, and internists, must accept rotating night shifts, which are considered a risk factor for DM [9]. Heavy workloads and rotating night shifts may contribute to poor lifestyles with insufficient physical activity and unhealthy diets, both of which are also risk factors for developing DM [10]. Searches in PubMed and Google Scholar for studies on the risk for DM in physicians were not found after searching for the key words “physician” and “diabetes mellitus.” Thus, we conducted this research to determine the risk for DM among physicians in Taiwan.

## Methods

### Data sources

Two sub-datasets of the National Health Insurance Research Database (NHIRD) were used for the current study, namely, the 2010 Registry for Medical Personnel and the Longitudinal Health Insurance Database 2000 (LHID 2000). The 2010 Registry for Medical Personnel includes information about the specialty, work area, license date, hospital level, types of employment, and encrypted identification number of physicians, nurses, pharmacists, and other healthcare providers, which can be linked to claims data [11]. The LHID 2000 features the claims data of 1 million beneficiaries (4.34% of the total population) who were randomly selected from the NHIRD [12]. The NHIRD is derived from the Taiwan National Health Insurance Program, a universal healthcare system that covers nearly 100% of the country’s population [11]. The database of this program contains registration files and original claims data for reimbursement [11]. Large computerized databases derived from this system by the National Health Insurance Administration, Ministry of Health and Welfare, Taiwan, and maintained by the National Health Research Institutes, Taiwan, are provided to scientists in Taiwan for research purposes [11].

### Study design

**Fig. 1:**
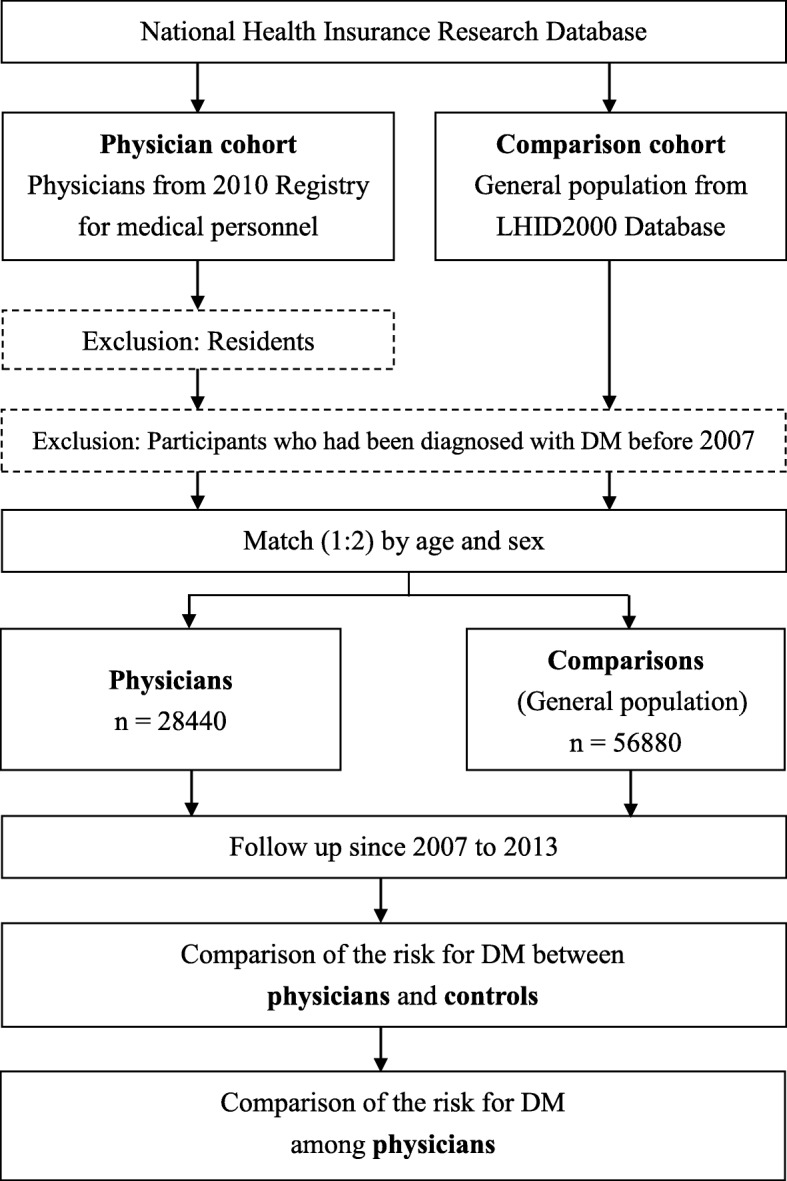
Flowchart of this study. Longitudinal Health Insurance Database, LHID; diabetes mellitus, DM

We identified all physicians from the 2010 Registry for Medical Personnel and comparisons (i.e., general population after excluding healthcare providers) from the LHID 2000 registered in 2010 by matching age and sex at a ratio of 1:2 for this study (Fig. 1). Residents were excluded because they did not have a specialty board and, therefore, cannot be categorized into a physician specialty for comparison. Residents also tend to have short experiences as physicians, which means their work may not completely reflect the effect of occupational exposure. DM was defined by the International Classification of Diseases, Ninth Revision, Clinical Modification (ICD-9-CM) codes of 250 in at least one hospitalization or at least three ambulatory care events. Participants who had been diagnosed with DM before 2007 were excluded. Comorbidities were defined as hypertension (ICD-9-CM: 401–405), hyperlipidemia (ICD-9-CM: 272), hyperuricemia (ICD-9-CM: 274), coronary artery disease (ICD-9-CM: 410–414), congestive heart failure (ICD-9-CM: 428), hyperthyroidism (ICD-9-CM: 242), hypothyroidism (ICD-9-CM: 243–244), and polycystic ovary syndrome (ICD-9-CM: 256.4) in at least one hospitalization or at least three ambulatory care events between 2007 and 2013. We compared the risk of DM between physicians and comparisons, as well as among physician subgroups, in terms of specialty, age, and sex by following up the participants’ medical histories since January 1, 2007 to December 31, 2013. We classified the physicians into the following six specialties for the analysis: internal medicine, surgery, obstetrics and gynecology (Ob/Gyn), pediatrics, emergency medicine, family medicine, and other specialties.

### Ethics statement

This study was conducted according to the Declaration of Helsinki. The Institutional Review Board of Chi-Mei Medical Center approved of this study and waived the need for informed consent from participants because the dataset consists of de-identified data. This waiver does not affect the rights and welfare of the participants.

### Statistical analysis

We used the chi-square test for categorical variables and the independent *t*-test for continuous variables to compare the demographic characteristics between the two groups. Conditional logistic regression was used to compare the risk for DM between physicians and comparisons. Comparisons of risks for DM among specialty, age, and sex subgroups in physicians were performed by unconditional logistic regression with adjustment of age, sex, hypertension, hyperlipidemia, hyperuricemia, coronary artery disease, congestive heart failure, hyperthyroidism, hypothyroidism, and polycystic ovary syndrome. SAS (version 9.4 for Windows, SAS Institute, Inc., Cary, NC, USA) was used for all analyses in this study, and significance was set at 0.05 (two-tailed).

## Results

We identified 28,440 physicians and 56,880 age- and sex-matched comparisons for the current study (Table 1). The mean age of both physicians and comparisons was 46.5 ± 10.9 years. Males made up 85.3% of the participants in both groups. Compared with the general population, more physicians had hypertension (20.0% vs. 18.9%, *p* < 0.001) and hyperlipidemia (19.3% vs. 13.7%, *p* < 0.001) but fewer physicians had coronary artery disease (5.4% vs. 6.0%, *p* < 0.001) and congestive heart failure (0.5% vs. 1.1%, *p* < 0.001).

**Table 1:** Age, sex, and comorbidities of physicians and comparisons (general population)

Characteristic	Physicians (*n* = 28,440)	Comparisons (*n* = 56,880)	*p-*value
Age (years)	46.5 ± 10.9	46.5 ± 10.9	> 0.999
Age (years)			
< 35	3985 (14.0)	7970 (14.0)	> 0.999
35–49	14,279 (50.2)	28,558 (50.2)	
50–64	8497 (29.9)	16,994 (29.9)	
≥ 65	1679 (5.9)	3358 (5.9)	
Sex			
Male	24,258 (85.3)	48,516 (85.3)	> 0.999
Female	4182 (14.7)	8364 (14.7)	
Comorbidity			
Hypertension	5673 (20.0)	10,761 (18.9)	< 0.001
Hyperlipidemia	5496 (19.3)	7790 (13.7)	< 0.001
Hyperuricemia	2101 (7.4)	4230 (7.4)	0.796
Coronary artery disease	1532 (5.4)	3385 (6.0)	< 0.001
Congestive heart failure	147 (0.5)	645 (1.1)	< 0.001
Hyperthyroidism	128 (0.5)	216 (0.4)	0.127
Hypothyroidism	260 (0.9)	482 (0.9)	0.322
Polycystic ovary syndrome	58 (0.2)	90 (0.2)	0.130

Data are number (%) or mean ± SD

Conditional logistic regression analyses with adjustment for hypertension, hyperlipidemia, hyperuricemia, coronary artery disease, congestive heart failure, hyperthyroidism, hypothyroidism, and polycystic ovary syndrome showed that physicians had a lower risk for DM than the general population (adjusted odds ratio [AOR]: 0.75; 95% confidence interval [CI]: 0.68–0.82) (Table 2). Stratified analyses according to age showed a lower risk for DM in physicians in the subgroups of 35–49 years (AOR: 0.68; 95% CI: 0.57–0.81) and 50–64 years (AOR: 0.74; 95% CI: 0.65–0.85). Although male physicians had a lower risk for DM than male comparisons (AOR: 0.74; 95% CI: 0.67–0.82), the same trend was not observed between female physicians and comparisons (AOR: 0.95; 95% CI: 0.64–1.42).

**Table 2:** Comparison of DM risk between physicians and comparisons (general population) by conditional logistic regression

	Number (%)	OR (95% CI)	AOR (95% CI)^a^
Overall analysis			
Physicians	1339 (4.7)	0.73 (0.69–0.78)	0.75 (0.68–0.82)
Comparisons	3575 (6.3)	1 (reference)	1 (reference)
Stratified analysis			
Age subgroup			
< 35 years			
Physicians	50 (1.3)	0.85 (0.61–1.19)	0.82 (0.48–1.41)
Comparisons	117 (1.5)	1 (reference)	1 (reference)
35–49 years			
Physicians	426 (3.0)	0.68 (0.60–0.76)	0.68 (0.57–0.81)
Comparisons	1241 (4.4)	1 (reference)	1 (reference)
50–64 years			
Physicians	660 (7.8)	0.73 (0.66–0.80)	0.74 (0.65–0.85)
Comparisons	1768 (10.4)	1 (reference)	1 (reference)
≥ 65 years			
Physicians	203 (12.1)	0.89 (0.75–1.06)	0.96 (0.68–1.36)
Comparisons	449 (13.4)	1 (reference)	1 (reference)
Sex			
Male			
Physicians	1251 (5.2)	0.73 (0.69–0.78)	0.74 (0.67–0.82)
Comparisons	3330 (6.9)	1 (reference)	1 (reference)
Female			
Physicians	88 (2.1)	0.70 (0.55–0.90)	0.95 (0.64–1.42)
Comparisons	245 (2.9)	1 (reference)	1 (reference)

*DM* diabetes mellitus, *OR* odds ratio, *AOR* adjusted odds ratio, *CI* confidence interval

^a^Adjusted by hypertension, hyperlipidemia, hyperuricemia, coronary artery disease, congestive heart failure, hyperthyroidism, hypothyroidism, and polycystic ovary syndrome

Emergency physicians (AOR: 2.21; 95% CI: 1.44–3.40) and surgeons (AOR: 1.26; 95% CI: 1.05–1.52) had a higher risk for DM than other specialists (Table 3). Older and male physicians had a higher risk than their counterparts.

**Table 3:** Comparison of DM risk among physician specialties by unconditional logistic regression

	Number (%)	OR (95% CI)	AOR (95% CI)^a^
Specialty			
Internal medicine	212 (3.8)	0.80 (0.68–0.93)	0.92 (0.78–1.09)
Surgery	168 (5.9)	1.25 (1.05–1.49)	1.26 (1.05–1.52)
Ob/Gyn	91 (5.2)	1.09 (0.87–1.37)	0.95 (0.75–1.20)
Pediatrics	90 (3.5)	0.73 (0.59–0.92)	0.98 (0.77–1.24)
Emergency medicine	26 (4.8)	1.01 (0.67–1.51)	2.21 (1.44–3.40)
Family medicine	132 (6.4)	1.36 (1.12–1.65)	1.20 (0.97–1.48)
Other specialties	620 (4.7)	1 (reference)	1 (reference)
Age subgroup			
< 35	50 (1.3)	1 (reference)	1 (reference)
35–49	426 (3.0)	2.42 (1.80–3.25)	1.73 (1.28–2.35)
50–64	660 (7.8)	6.63 (4.96–8.86)	3.40 (2.50–4.62)
≥ 65	203 (12.1)	10.82 (7.90–14.83)	4.43 (3.14–6.24)
Sex			
Male	1251 (5.2)	2.53 (2.03–3.15)	1.32 (1.04–1.68)
Female	88 (2.1)	1 (reference)	1 (reference)

*DM* diabetes mellitus, *AOR* adjusted odds ratio, *CI* confidence interval, *HTN* hypertension

^a^Adjusted by age, sex, hypertension, hyperlipidemia, hyperuricemia, coronary artery disease, congestive heart failure, hyperthyroidism, hypothyroidism, and polycystic ovary syndrome

## Discussion

The current study found that the risk for DM in physicians was lower than that in the general population. Stratified analyses showed lower risks in the age subgroups of 35–49 years and 50–64 years and in the male population. Emergency physicians and surgeons had a higher risk for developing DM than other specialists. In physicians, male sex and older age were risk factors for DM.

An explanation for the lower risk for DM observed among physicians in comparison with the general population is that, despite heavier workloads and the related poor lifestyle, the former have better medical knowledge, higher disease awareness, and easier healthcare access than the latter; these benefits may mitigate the risk for DM among physicians. The current result is also compatible with previous studies in Taiwan that showed that, despite physicians having higher risks for hypertension, hyperlipidemia, migraine, and herniated intervertebral disc than the general population [13–16], the former were also less vulnerable to major and life-threatening diseases, including cardiovascular, cancer, and severe sepsis than the latter [13, 17–19].

The finding of lower risks for DM in the subgroups of age 35–64 years and male physicians suggests that age and sex are effect modifiers. Although a better medical background may be a protective factor for physicians, younger physicians (< 35 years) have shorter occupational exposures and may not reflect the true status of a physician. The current result is compatible with a previous study that reported that physicians have a lower risk for stroke than the general population; however, this lower risk was not found in physicians younger than 35 years [17]. Older physicians (≥65 years) may have less disease awareness and medical access and more comorbidities, which may decrease the benefit of a better medical background [19]. Why a lower risk for DM was found only in male physicians is not clear. A previous study showed a similar finding, i.e., all-cancer risk was lower in male physicians than in the male general population; however, female physicians did not show this trend [18]. Further studies on this disparity are necessary.

The finding that emergency physicians and surgeons are at higher risk for DM than other physician specialties may be related to the heavier work stress and frequent night shifts of the former. A stressful work environment may increase the risk of type 2 DM [20]. A population-based study previously reported that participants with high job stress have a 45% higher risk for developing type 2 DM than those with low job stress [20]. According to the SESMAT study in France, emergency physicians accumulate more stress than other physicians [21]. Work-family conflict and quality of teamwork are independent predictors of stress [21]. A burnout and satisfaction research showed that emergency physicians have a 3-fold higher burnout rate compared with those of other specialties [22]. A study recruiting 2860 participants in Japan reported that shift workers had a higher risk for DM than fixed daytime workers [23]. Another study recruiting 10 participants who had undergone a 10-day laboratory protocol reported that circadian misalignment increases serum glucose [24]. In Nurses’ Health Studies I and II, extended periods of rotating night-shift work among nurses resulted in a significant increase in risk for type 2 DM [9]. In addition, rotating night-shift workers may develop unhealthy behaviors, including smoking and irregular mealtimes, which may also increase the risk for DM [9].

Among physicians, older age and male sex were associated with increased risk for DM, compatible with previous studies. A nationwide study in Taiwan reported that the incidence of DM was higher in men, especially in the 20–59-year-old age group, then in women [2]. Impaired fasting glucose is significantly more common in men than in women [25]. One possible reason for this difference is that men tend to have lower hepatic sensitivity to insulin and, therefore, generally higher fasting levels of plasma glucose than women [26].

The current study presents two strengths: it is the first study to compare the risk for DM between physicians and the general population and the study features a nationwide design. Several limitations must also be acknowledged. First, some important risk factors of DM, including body mass index, life style, and family history of DM, were not considered in this work. However, we adjusted for hypertension and hyperlipidemia, which may serve as substitutes for body mass index and lifestyle. Second, data on occupational exposure, including workload, stress, and night shifts, were not available, and this lack of information may affect our comparisons among physician specialties. Third, the discrepancy about lower risk of DM but higher prevalence of hypertension and hyperlipidemia in the physicians than in the general population could not be explained in the present study. Because it is beyond the scope of this study, further research about this issue is warranted in the future. Fourth, a six-year follow-up may not be long enough to confirm our findings; a longer research period may be necessary. Finally, the results may not be generalized to other nations due to differences in race, health care systems, and cultures.

## Conclusion

The current study revealed that, despite their heavy workloads, physicians are at lower risk for DM than the general population. This finding could be explained by the former having better medical knowledge, higher disease awareness, and easier healthcare access, which may compensate for the effect of heavy workloads, than their general-population counterparts. Comparisons among physicians revealed that emergency physicians and surgeons are at higher risk for developing DM than other specialists, which may be explained by these two specialties experiencing heavier work stresses and more frequent night shifts than their counterparts in other specialties. In addition, male sex and older age were predictive of DM risk in physicians. The results of this work provide an important reference for future efforts to improve occupational health among physicians, especially those in high-risk specialties, such as emergency physicians and surgeons.

## Data Availability

Data are available from the National Health Insurance Research Database (NHIRD), which is published by the Taiwan National Health Insurance Bureau. Due to legal restrictions imposed by the government of Taiwan in relation to the Personal Information Protection Act, data cannot be made publicly available. Requests for data can be sent as a formal proposal to the NHIRD (http://nhird.nhri.org.tw).

## Abbreviations

AOR: Adjusted odds ratio
CI: Confidence interval
DM: Diabetes mellitus
ICD-9-CM: International Classification of Diseases, Ninth Revision, Clinical Modification
LHID: Longitudinal Health Insurance Database
NHIRD: National Health Insurance Research Database
Ob/Gyn: Obstetrics and gynecology
